# Phyllosphere symbiont promotes plant growth through ACC deaminase production

**DOI:** 10.1038/s41396-023-01428-7

**Published:** 2023-06-01

**Authors:** Johannes B. Herpell, Ajtena Alickovic, Bocar Diallo, Florian Schindler, Wolfram Weckwerth

**Affiliations:** 1 Molecular Systems Biology Division, Department of Functional and Evolutionary Ecology, University of Vienna, Djerassiplatz 1, 1030 Vienna, Austria; 2 Vienna Metabolomics Center, University of Vienna, Djerassiplatz 1, 1030 Vienna, Austria

**Keywords:** Microbiology, Environmental microbiology, Microbial ecology, Symbiosis, Plant sciences

## Abstract

Plant growth promoting bacteria can confer resistance to various types of stress and increase agricultural yields. The mechanisms they employ are diverse. One of the most important genes associated with the increase in plant biomass and stress resistance is *acdS*, which encodes a 1-aminocyclopropane-1-carboxylate- or ACC-deaminase. The non-proteinogenic amino acid ACC is the precursor and means of long-distance transport of ethylene, a plant hormone associated with growth arrest. Expression of *acdS* reduces stress induced ethylene levels and the enzyme is abundant in rhizosphere colonizers. Whether ACC hydrolysis plays a role in the phyllosphere, both as assembly cue and in growth promotion, remains unclear. Here we show that *Paraburkholderia dioscoreae* Msb3, a yam phyllosphere symbiont, colonizes the tomato phyllosphere and promotes plant growth by action of its ACC deaminase. We found that *acdS* is required for improved plant growth but not for efficient leaf colonization. Strain Msb3 readily proliferates on the leaf surface of tomato, only occasionally spreading to the leaf endosphere through stomata. The strain can also colonize the soil or medium around the roots but only spreads into the root if the plant is wounded. Our results indicate that the degradation of ACC is not just an important trait of plant growth promoting rhizobacteria but also one of leaf dwelling phyllosphere bacteria. Manipulation of the leaf microbiota by means of spray inoculation may be more easily achieved than that of the soil. Therefore, the application of ACC deaminase containing bacteria to the phyllosphere may be a promising strategy to increasing plant stress resistance, pathogen control, and harvest yields.

## Introduction

Plants live in association to a diverse set of microorganisms, termed the plant microbiota, which influence plant phenotype and fitness [1–3]. Through interactions with the plant, or other members of the microbiota, they can provide beneficial functions to the plant host, such as promotion of plant growth or resistance to biotic and abiotic stress factors [4–8]. As such, exploiting these functions presents a viable alternative to environmentally harmful pesticides and fertilizers. Correctly employing plant growth promoting bacteria (PGPB) in biologicals could lead to more sustainable practices in agriculture [9, 10]. Deciphering the mechanisms underpinning these beneficial services is, therefore, of utmost importance for their safe application.

There is a variety of different mechanisms that PGPB employ to facilitate plant growth and health. These include indirect mechanisms like priming host immunity to induce systemic resistance [11] or microbiome shifts [12], and the suppression of bacterial or fungal phytopathogens [6] as well as direct effects such as increasing the supply of nutrients [13, 14] or modulating plant hormone pools [1, 15–17]. The later comprises biosynthesis [18] or degradation [1] of auxins and the regulation of ethylene levels [15–17]. Ethylene is, in high concentrations, associated with growth arrest and senescence [19–21] but lower levels can lead to non-inhibited and even growth promoted phenotypes [20]. Lowering “stress ethylene” has been demonstrated to be a very efficient and reliable way to facilitate plant growth under varying environmental conditions [16, 22]. Root associated bacteria (and fungi) hydrolyze the non-proteinogenic amino acid 1-aminocyclopropane-1-carboxylate (ACC) [17], which is the direct precursor of ethylene [23]. Ethylene biosynthesis is largely controlled at the level of ACC production. Lower availability of ACC leads to a reduction in its transport rates and lower overall ethylene production in the plant [24]. ACC synthesis by ACC synthases is differentially regulated at the transcriptional level in an organ-, tissue- or cell-type specific manner throughout the entire plant [25].

ACC is found in root exudates and is, as a chemoattractant, considered an assembly cue for plant growth promoting rhizobacteria (PGPR) [26–28]. However, information regarding the role that microbial ACC hydrolysis plays in the phyllosphere is scarce, even though various isoforms of the ACC synthase are expressed in young leaves and in the epidermal layers of the hypocotyl [24].

Leaf colonization is an important trait of PGPB in respect to agricultural applicability within biologicals [29]. Spraying the plant and thereby manipulating the phyllosphere microbiota may be more easily and rapidly achieved than that of soil and rhizosphere microbiota. To the best of our knowledge, ACC hydrolysis and its growth promoting effect have only been mechanistically validated for rhizosphere colonizers, but not for leaf colonizers. Here we report a plant-beneficial interaction between *Paraburkholderia dioscoreae* strain Msb3 [30] (henceforth Msb3) and *Lycopersicum esculentum* cv. Moneymaker (henceforth tomato). Msb3 is a plant growth promoting bacterium that has been isolated from the leaves of *Dioscorea bulbifera*, a tropical yam [31]. We set out to understand the mechanism through which Msb3 facilitates growth and which host tissues it colonizes. We hypothesize that phyllosphere colonization is a general strategy of Msb3 and that plant growth promotion (PGP) there is, at least partially, achieved by action of its ACC deaminase. Additionally, we postulate that microbially mediated depletion of the ACC pool in the above ground tissues of plants can be another major contributor to PGP by plant associated bacteria.

## Results

### Msb3 readily colonizes the tomato phyllosphere and promotes its growth

In order to understand the growth facilitative effects of Msb3 we designed several experiments to a) evaluate and reproduce the growth promoting properties of Msb3 on a tomato host, b) to test whether Msb3 also colonizes the leaves of hosts other than *D. bulbifera*, its source of isolation [31], and c) to investigate the persistence and stability of the association between the bacteria and their host.

**Fig. 1:**
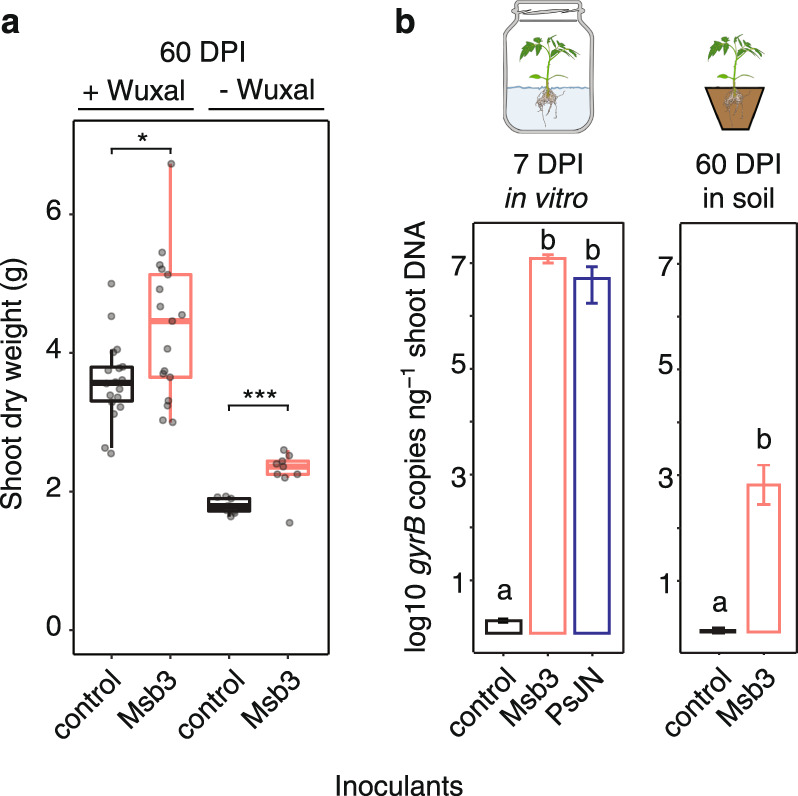
Tomato colonization and growth promotion. **a** Tomatoes were grown for 60 days in pots after inoculation with either heat killed or live Msb3. Two thirds of the plants were fertilized with a commercial plant fertilizer (+Wuxal) while one third did not receive any fertilization (−Wuxal). Depicted is the dry weight of the shoots 60 DPI (*n* = 18, 17, 9, 9). Significance was determined between treatments via a two-sided Students’ T-test; the stars correspond to the level of significance. **b** In two contrasting plant cultivation systems *P. dioscoreae* strain Msb3 can be found on above ground tissues. Both panels display species specific copy numbers of the single copy marker gene *gyrB* detected through qPCR. After seven days within a gnotobiotic growth system strain Msb3 is very abundant (*n* = 5). *P. phytofirmans* strain PsJN was included as a positive control (*n* = 5). Negative controls were non-inoculated tomato seedlings (*n* = 5). Msb3 could also be detected on the topmost tomato leaves of plants grown in soil 60 days post inoculation (DPI) (*n* = 4). Controls were inoculated with heat killed bacteria (*n* = 4). Significance was determined within treatments via ANOVA; letters correspond to a Tukey *post hoc* test.

To evaluate the growth promoting behavior of the strain we conducted plant growth assays in soil. We inoculated tomato seedlings with live or heat killed Msb3 (3 × 10^6^ CFUs ml^−1^) and measured plant growth after 60 days with and without fertilization (Fig. 1). We were able to show that Msb3 inoculated plants grew significantly heavier than the control in both non-fertilized pots as well as pots fertilized with the commercial plant fertilizer Wuxal (Fig. 1a). On average, dry weight of inoculated plants increased as much as 28% relative to the control under a non-fertilized regime and up to 21% under a fertilized regime.

We also quantified bacterial abundance on the leaves of 60-day old plants. The youngest, topmost leaves next to the apical meristem were sampled to make sure that only bacteria were considered that actively spread to freshly emerging leaves. Their abundance was estimated via qPCR of the single copy marker gene *gyrB*. We designed species specific primers that only target strains of *P. dioscoreae*. We found that 60 days post inoculation (DPI) of small plantlets the youngest leaves of mature plants were still consistently positive for Msb3 colonization. On average we detected 600 ± 430 copies of the Msb3 *gyrB* per ng of DNA extracted from those leaves (Fig. 1b). While the amount is not large it may efficiently function as inoculum to sustain larger colony formation on the expanding leaf. We did not detect any trace of Msb3 colonization on control plants. To enable a faster pace for subsequent investigations we experimented with different settings for tomato growth in vitro that would lead to comparable results. We came up with an in vitro growth system for tomato in Murashige & Skoog (MS) agar (see Methods), in which plantlets could be grown for up to four weeks. During the first two weeks plants develop normally. Subsequently, they retain a dwarfish phenotype that looks healthy otherwise. We inoculated plants with Msb3 and sampled the shoots of the plants after one week to quantify bacterial load in the same manner as described before. As positive control we included *Paraburkholderia phytofirmans* PsJN (henceforth PsJN), a well-known systemic plant colonizer [32–34], for which we designed another set of species-specific primers targeting the same region within the *gyrB* gene. As negative control we used uninoculated seedlings because DNA from heat killed cells may have confounded the qPCR analysis. Seven DPI both strains had reached densities as high as 10^7^ gene copies ng^−1^ DNA (Fig. 1b), which exceeds the number of cells possibly present due to inoculation by at least an order of magnitude (see Methods).

### Msb3 actively divides on the leaf surface

**Fig. 2:**
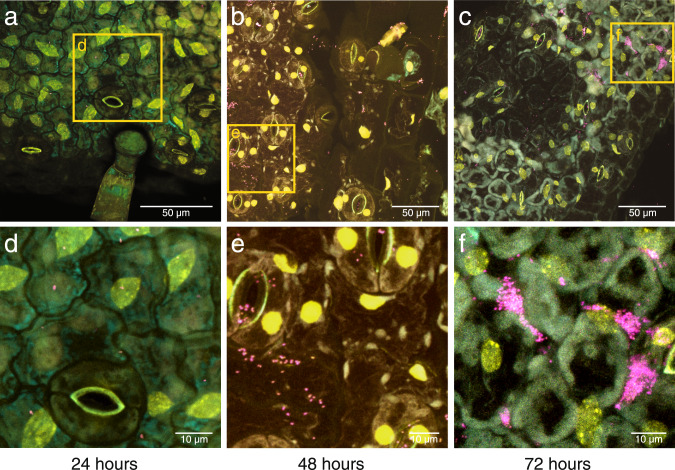
Microscopic analysis of Msb3 population dynamics after leaf inoculation. Visualization of bacteria by DOPE-FISH/CLSM microscopy. The FISH probe Burkho was applied to leaves of tomato. The lower epidermis is depicted at different timepoints: **a** 24, **b** 48 and **c** 72 hours post inoculation with a highly diluted bacterial suspension. Plant autofluorescence was visualized through the cyan channel, the Burkho probe through the magenta channel and the nucleic acid stain SYBR-Safe through the yellow channel. Panels **a**–**f** all show overlays of all three channels. Panels **d**, **e** and **f** show high-resolution images of the sections highlighted with a yellow frame in **a**, **b** and **c**, respectively.

To explain the copy numbers detected on the leaves, we hypothesized that Msb3 was able to actively divide on or within tomato leaves. To test this, we inoculated seedlings with a highly diluted bacterial suspension (OD_600_ of 0.001) and performed microscopic analyses on a confocal laser scanning microscope (CLSM) after fluorescent in situ hybridization (FISH) with the probe Burkho [35]. The plants were washed before hybridization to remove cells that were only present due to the inoculation rather than attached to the leaf surface. We performed the experiment in a timeseries, sampling plants after 24 hours, 48 hours as well as 72 hours post inoculation. The pattern we observed confirmed our hypothesis (Fig. 2). We detected Msb3 on the leaf surface, namely on the lower epidermis, in all inoculated samples but not in controls. After 24 hours there were only a few individual cells attached to the lower side of the leaf lamina (Fig. 2a, d). After 48 hours clusters of cells emerged (Fig. 2b, e) and after 72 hours these clusters had further matured (Fig. 2c, f). From these observations we concluded that Msb3 was actively dividing in the phyllosphere.

### Msb3 is most abundant in the phyllosphere

**Fig. 3:**
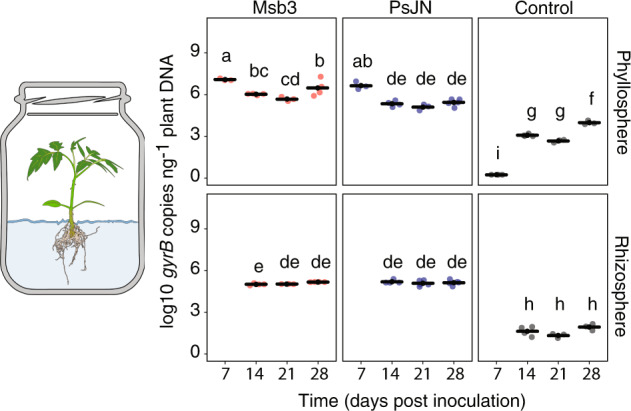
Quantitative analysis of Msb3 and PsJN population dynamics on different tissues via qPCR. Tomato plants were grown in vitro in gnotobiotic systems. One third of the plants was inoculated with Msb3 (left panels), one third with PsJN (middle panels) and one third was not inoculated functioning as control (right panels). Each week (x-axes) some plants were harvested, divided into phyllosphere (upper panels) and rhizosphere (lower panels) sections and submitted to DNA extractions. qPCRs were performed on the single copy marker gene *gyrB* with different, species specific primers for both Msb3 and PsJN. Control samples were subjected to amplification with Msb3 specific primers. Shown are the log10 copy numbers per ng of total DNA (y-axes). Significance was determined between all timepoint and treatments via ANOVA; letters correspond to a Tukey *post hoc* test.

We conducted another set of in vitro experiments. Seedlings were inoculated with Msb3, PsJN or not at all. Over a period of four weeks we took a set of samples once a week and quantified *gyrB* gene copies in the DNA extracts from either the shoot or the root system. This enabled us to compare the growth behavior of Msb3 and PsJN in the phyllosphere with that in the rhizosphere. Msb3 was significantly more abundant in the phyllosphere than in the rhizosphere at any given point in time (Fig. 3). The abundance of both strains in the phyllosphere, relative to plant DNA, decreased drastically during the second week and slightly during the third week of growth. During these periods the plant shoot extended with maximum velocity. 21 DPI the bacterial abundance on the leaf stabilized and increased again. Similarly, this effect correlated with the beforementioned growth arrest after weeks inside small containers. 28 DPI Msb3 remained significantly more abundant in the phyllosphere than PsJN with an average count above 5 × 10^6^ *gyrB* copies ng^−1^ DNA for Msb3 and around 3 × 10^5^ copies for PsJN.

Although root extension velocity displayed similar oscillations to those in the shoot, the relative bacterial abundance in the rhizosphere remained stable throughout. (Fig. 3). Controls in the rhizosphere did not indicate any signs of contamination or off-target amplification.

In phyllosphere control samples Msb3 was not detectable 7 DPI, but some copies were detected after two weeks (14 DPI). The highest values amounted to 10^4^ *gyrB* copies ng^−1^ DNA. qPCR melting curves, however, were homogeneous and congruent to those of the Msb3 *gyrB* amplicon, indicating contamination of the in vitro cultures with Msb3 and not off-target amplification of other bacteria. The parafilm that was used to seal the culture jars became brittle after a week, which could have allowed bacteria to penetrate it.

**Fig. 4:**
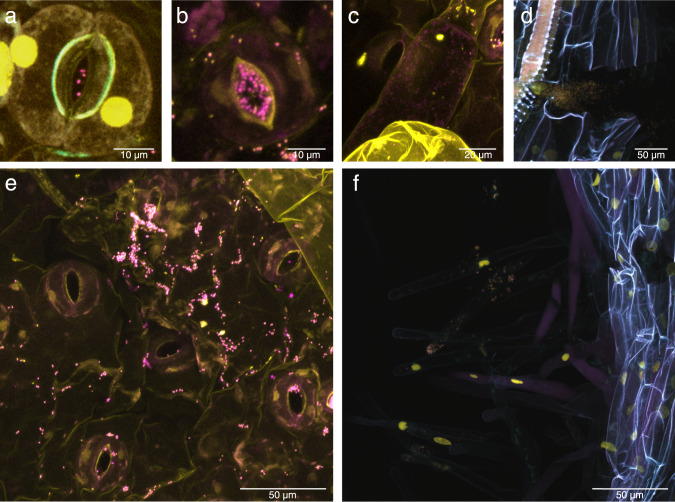
Colonization patterns of strain Msb3 on tomato. Visualization of bacteria by DOPE-FISH/CLSM microscopy. Plant autofluorescence was visualized in cyan, signals representing the Burkho probe in magenta and that of the nucleic acid stain SYBR-Safe in yellow. **a**, **d** and **f** are composite images of all three channels, **b**, **c** and **e** are overlays of the magenta and the yellow channel only, as the yellow channel was usually sufficient to visualize epidermal autofluorescence in leaf samples. **a**, **b**, **c** and **e** are images of the lower leaf epidermis, **d** and **f** of roots. **a** and **b** show stomata colonized by Msb3, **c** a trichome. **d** shows a root crack filled with labelled bacteria, **f** shows a non-colonized 1st degree side root with root hairs that are colonized at the tips.

To shed light on the ecology of strain Msb3 and to understand which sites on the plant it colonizes we analyzed several samples from those timepoints through FISH analysis. The technique was conducted in the same manner as described before. We observed several conserved patterns (Fig. 4). Strain Msb3 often colonized stomata on the leaf but it was rarely found deep inside the substomatal space (Fig. 4a, b). We almost exclusively found Msb3 on the leaf surface, never in the endosphere, except occasionally inside of trichomes (Fig. 4c). On the lower epidermis Msb3 was frequently detected in large clusters (Figs. 2b, e, c, f and 4e). These usually formed along the grooves between epithelial cells (Fig. 4e). In the root system we observed transient colonization of the tips of root hairs but not of the main root (Fig. 4f). Bacteria seemed to cluster inside the root, in cracks, only if the root was wounded (Fig. 4d).

### Plant growth promotion of Msb3 is mainly achieved through manipulation of ethylene biosynthesis

We developed strategies to investigate the molecular mechanism through which Msb3 promotes the growth of its tomato host. Genomic analysis has revealed that Msb3 carries several genes associated with facilitative strategies [31]. We have previously hypothesized about the roles of nitrogen fixation in facultative symbiotic interactions as a further source of nutrients [31] as well as plant hormone regulation through the ACC deaminase gene *acdS*. Both pathways have been studied in root nodulating and rhizosphere colonizing bacteria, respectively [5, 17, 22, 36, 37]. However, the roles these pathways play in phyllosphere colonizers are largely unknown. We generated mutant lines of Msb3, knocking out *acdS* or *nifH* through an allelic exchange system based on homologous recombination [38]. Knocking out *nifH*, the marker gene for nitrogen fixation, had no effect on both plant colonization and growth promotion properties (data not shown). This is in line with our finding that nitrogen fixation does not occur within our in vitro setting based on ^15^N_2_ labelling experiments (Supplementary Fig. 1). We conclude therefore that supply of nitrogen does not seem to contribute to the plant growth promoting effect we observe.

**Fig. 5:**
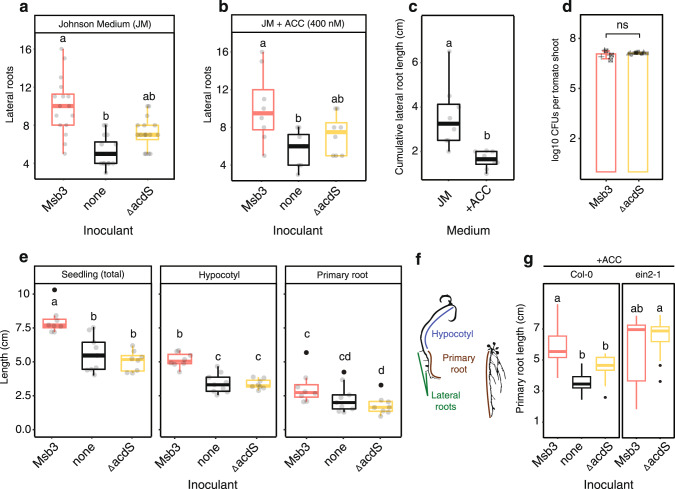
Phenotypic changes in tomato and *Arabidopsis* in response to Msb3 or Msb3ΔacdS. **a** Number of 1st degree side roots of tomato seedlings grown in Johnson medium (JM) 7 DPI with Msb3 or the *acdS* deficient mutant of Msb3 and a non-inoculated control (*n* = 16). Significance was determined within each treatment via ANOVA; letters correspond to a Tukey *post hoc* test. **b** number of 1st degree lateral roots of tomato seedlings grown in Johnson medium (JM) that contained 400 nM of ACC with the same conditions and treatments as in **a** (*n* = 8). Significance was determined within each treatment via ANOVA; letters correspond to a Tukey *post hoc* test. **c** Cumulative length of the 1st degree side roots of non-inoculated samples in JM or JM containing 400 nM of ACC. Significance was determined within each treatment via ANOVA; letters correspond to a Tukey *post hoc* test. **d** CFUs recovered from whole tomato shoots inoculated with fluorescently labelled derivative strains of either Msb3 wt or the *acdS* deficient mutant. Two Msb3 derivative strains (*n* = 3 each) make up the red bar, three Msb3ΔacdS derivative strains (*n* = 5 each) make up the yellow bar. The jittered shapes correspond to the respective derivative mutant: pluses: Msb3::eGFP2; checked boxes: Msb3::mScarlet; circles: Msb3ΔacdS::eGFP2.1; triangles: Msb3ΔacdS::eGFP2.2; squares: Msb3ΔacdS::mScarlet. **e** Lengths of different phenotypic parameters of tomato seedlings grown in Johnson medium (JM) 7 DPI with Msb3 or the *acdS* deficient mutant of Msb3 and a non-inoculated control (*n* = 8). Significance was determined within each treatment and between all parameters via ANOVA; letters correspond to a Tukey *post hoc* test. **f** Binarized images of representative seedlings of tomato (left) and *Arabidopsis* (right) to illustrate which phenotypic parameters were considered. **g** Primary root elongation of *Arabidopsis* seedlings, either the *Arabidopsis* wt Col-0 or the ethylene insensitive mutant line ein2-1, grown with RGI-inducing hormonal treatments (100 nM ACC), individually (none) or with Msb3 or the *acdS* deficient mutant of Msb3 (*n* = 8). Significance was determined within each treatment via ANOVA; letters correspond to a Tukey *post hoc* test.

The ACC deaminase knockout, however, attenuated the plant growth promoting effect of strain Msb3. *P. dioscoreae* Msb3 Δ*acdS* (Msb3ΔacdS) can no longer grow on ACC as carbon or nitrogen source (Supplementary Fig. 2) and plants inoculated with it no longer produce the same number of lateral roots as Msb3 wildtype (wt) inoculated plants (Fig. 5a). The number of lateral roots is not affected by addition of ACC to the growth medium, indicating that stimulation of lateral root formation in tomato is not exerted through ACC hydrolysis in the rhizosphere (Fig. 5b). The length of the lateral roots, however, is affected by treatment with ACC (Fig. 5c). We concluded that ACC in the medium inhibits root extension but not lateral root formation. Msb3 dependent growth promotion leads to lateral root formation, which is not influenced by ethylene signaling in roots.

To gather more detailed information on plant phenotypic parameters we measured the lengths of our plantlets (Fig. 5e, f). We observed a significant increase in total seedling length of Msb3 inoculated seedlings compared to the non-inoculated control. This increase was also completely suppressed in Msb3ΔacdS treated plants, indicating it is ACC deaminase dependent (Fig. 5e). Furthermore, the most pronounced effect of Msb3 mediated ACC hydrolysis does not seem to be facilitation of primary root elongation but rather that of the hypocotyl (Fig. 5e).

To establish a causal relationship between Msb3 ACC deaminase activity and the manipulation of plant ethylene levels we conducted further experiments in *Arabidopsis thaliana* (*Arabidopsis*) taking advantage of the broad spectrum of mutant lines commercially available. We performed root growth inhibition (RGI) assays using ACC as RGI inducing agent. Primary root length of the *Arabidopsis* wt line Col-0 is heavily affected by ACC but inoculation of the medium with Msb3 leads to a suppression of RGI. In contrast, treatment with Msb3ΔacdS fails to significantly attenuate ACC induced RGI (Fig. 5f, g). The ethylene insensitive line ein2-1, deficient for a central regulator of ethylene perception and signal transduction, is not affected by ACC induced RGI. In the ein2-1 mutant there is no difference between primary root length of Msb3 or Msb3ΔacdS treated plants in ACC background (Fig. 5f). These results clearly point towards ethylene as elicitor of RGI and ACC hydrolysis as cause for its suppression.

### *acdS* deficiency does not affect capacity for leaf colonization

As ACC functions as a chemoattractant for PGPR [26, 27] we hypothesized that an *acdS* knock out could affect the capacity of Msb3 to colonize the leaf and thereby its PGP effect. To make sure that Msb3ΔacdS did not just display an impaired plant colonization phenotype we conducted re-cultivation assays to quantify CFUs as a proxy for active cells. To generate controlled conditions both Msb3 and Msb3ΔacdS were genetically modified to constitutively express fluorescent proteins and a kanamycin resistance cassette. Cells were re-cultivated from in vitro grown tomato seven DPI on M9 minimal medium with glucose as sole carbon source containing kanamycin to specifically select for our strains of interest. There were no indications that the ability of Msb3ΔacdS to colonize and survive on the tomato shoot were weakened (Fig. 5d, Supplementary Fig. 3). Both Msb3 and Msb3ΔacdS reached CFU counts as high as 10^7^ CFUs per tomato shoot, indicating that the ACC deaminase deficiency is the cause of the PGP suppression in Msb3ΔacdS and not its ability to colonize the shoot.

### *acdS*-dependent growth promotion in the presence of a native community attainable via foliar spray

**Fig. 6:**
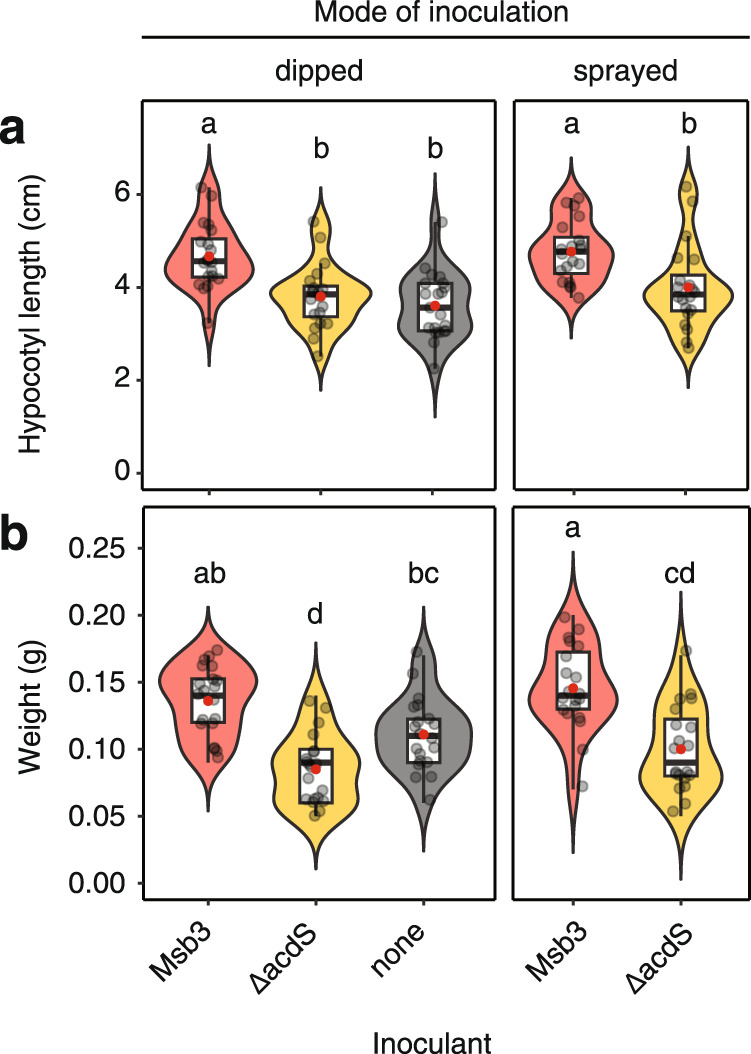
Effect of Msb3 and Msb3ΔacdS on tomato in the presence of a native microbial community and inoculated via foliar spray. **a** Hypocotyl length and **b**, weight of tomato seedlings grown in non-treated soil and exposed to the environment at all times. Measurements were taken 7 DPI with Msb3 or the *acdS* deficient mutant of Msb3 (ΔacdS). Two modes of inoculation were compared. Seedlings were either dipped into a suspension of bacterial cells (OD_600_ = 0.01) in 10 mM MgCl_2_ during transplanting or were sprayed with it after transplanting. Controls were dipped into an MgCl_2_ solution without inoculant (*n* = 20 for each mode of inoculation and each condition, respectively). Significance was determined within each treatment and between all parameters via ANOVA; letters correspond to a Tukey *post hoc* test. Red dots represent the mean.

To illustrate the potential for application of ACC deaminase-producing phyllosphere colonizers under non-sterile conditions, we conducted plant growth assays on non-gnotobiotic tomatoes grown in non-sterilized soil, exposed to the atmosphere for the duration of the experiment and watered with tap water. Seven-day-old seedlings were dipped into bacterial suspensions or a control solution and transferred to individual pots. After seven days of growth, we quantified both hypocotyl length as well as the weight of the seedlings (Fig. 6a, b). Results were in line with experiments conducted in vitro. Seedlings inoculated with Msb3 were significantly longer (Fig. 6a) and heavier (Fig. 6b) than those inoculated with Msb3ΔacdS or the control.

We added two more conditions to the experiment, changing the mode of inoculation. Instead of dipping the seedling into a bacterial suspension, we applied suspensions containing wt Msb3 or Msb3ΔacdS by spraying the shoot after transplanting. Pots and soil were covered to inoculate the shoot, specifically, and not the soil. The same plant parameters as for the dipped treatments were determined (Fig. 6a, b). Again, Msb3 inoculated seedlings significantly outperformed those sprayed with the *acdS* deficient strain or those not inoculated.

## Discussion

It has been widely accepted that ACC hydrolysis by ACC deaminase containing bacteria leads to promotion of plant growth [15–17, 22, 39]. A growing body of literature illustrates that bacteria that contain the structural protein sequence *acdS* or display ACC deaminase activity are abundant in the rhizosphere [40–43]. Clear evidence for the involvement of ACC deaminase in the promotion of plant growth comes from working with *acdS* mutant bacteria. There are several examples of this having been accomplished in root associated PGPB [39, 44, 45]. Although one study on its ACC deaminase activity was performed on *P. phytofirmans* PsJN, which we use here as a positive control for leaf colonization, it had only been detected in the canola rhizosphere at the time [39]. To the best of our knowledge, ACC hydrolysis by a phyllosphere colonizer has not been shown and experimentally validated through the development of an *acdS* deficient strain. *P. dioscoreae* Msb3 has been shown to colonize the phyllosphere of *Dioscorea bulbifera*, its source of isolation [31]. Here we have shown that Msb3 is a potent colonizer of the tomato phyllosphere as well (Figs. 1, 2, 3, 4).

Strain Msb3 also colonizes the root system to some degree (Figs. 3, 4). According to our microscopic observations, however, it can only transiently colonize the tips of root hairs (Fig. 4f) but not establish on the primary root surface or cortex (Fig. 4d). Additionally, it is by orders of magnitude more abundant in the phyllosphere (Fig. 3). The soil constitutes an important reservoir for phyllosphere bacteria [46, 47]. Survival in soil or in the rhizosphere may be an important feature for plant to plant transfer. Based on the metabolic versatility of strain Msb3 [30, 31] and usual exudate compositions [48], we believe that Msb3 can metabolize root exudates, which are commonly secreted through root hairs [49] and thereby sustain a small population within the tomato rhizosphere. However, its significant abundance and ability to divide on the leaf surface suggest that it is well adapted to life in the phyllosphere [47]. Therefore, a leaf associated lifestyle seems to be a general trait of Msb3. The genomic makeup that enables the colonization of the leaf surface specifically remains an open research question. For example, Msb3 does not carry genes for methylotrophy like many common leaf dwellers [50]. Instead it could make use of its extensive repertoire for carbon source utilization [31] to cope with the oligotrophic environment on the leaf surface. While diazotrophy could theoretically be a strategy for the acquisition of nitrogen, here we could show that the presence of *nifH* has no effect on the strains capacity to colonize this particular niche. Other strategies to cope with the harsh environmental conditions in the phyllosphere could include DNA and ROS-induced damage protection strategies via catalases and peroxidases and aggregate formation via quorum sensing [31, 47, 51, 52]. These features, in combination with its substantial plant growth promoting properties, make it a suitable candidate for application within biologicals that could be sprayed onto leaves.

We have also shown that Msb3 uses an ACC deaminase to promote growth of tomato (Figs. 5a, b, e and 6a, b) and to reduce ACC induced RGI in *Arabidopsis* (Fig. 5g). The *acdS* deficient strain loses much of its PGP activity. We could show that this process is related to ethylene perception in *Arabidopsis* but the data suggest that this is also true in tomato. Msb3 can use ACC as carbon and nitrogen source, which explains the RGI attenuating effect in ACC-treated *Arabidopsis*. The severe effect of Msb3 inoculation on hypocotyl length in tomato grown without inhibitory agent, however, can best be explained by ethylene pool manipulation via the tomato shoot. Indeed, we observe the same effects in plants that were inoculated by means of foliar spraying while covering the soil beneath (Fig. 6). These results are robust, considering that they are highly reproducible and that they occur not only under sterile conditions but also in the presence of a native microbial community (Figs. 1, 6).

ACC functions as chemoattractant for PGPB in the rhizosphere. For example, *acdS* is required for chemotaxis of *Pseudomonas* sp. UW4 towards ACC [27] and *acdS* overexpression can enhance its metabolism-dependent chemotactic response towards the rhizosphere [26]. In the present study, phyllosphere colonization is not affected by deletion of *acdS*, indicating that the gene is not required for chemotaxis towards the leaf or establishment of a population within the phyllosphere.

The current model of bacterially mediated ACC hydrolysis includes ACC transport into the rhizosphere and subsequently into the bacterial cytosol. ACC is likely transported via amino acid transporters [24, 53]. Shin et al. [53] have shown that ACC sprayed onto leaves can be imported via the amino acid transporter LHT1 in *Arabidopsis*. Database searches indicate that LHT1 is conserved in plants, therefore, an ACC transport system in the leaf seems to be in place. Like roots, leaves exude compounds depending on their nutritional state [54, 55]. This particular field of research is vastly understudied but some estimations suggest that, on average, the concentrations of amino acids and sugars in the apoplasm of leaf and stem tissue are in the range of 1–8 mM [54]. Not only do these results explain how microbial life on a leaf can be sustained, they also illustrate that amino acids like ACC can be readily exported to and imported from the leaf surface. This shows that the model for PGP through ACC deaminase containing rhizobacteria [15–17, 22] can also be applied to leaf colonizers. Furthermore, there is strong evidence that the epidermis is of primary importance in the control of cell expansion in the shoot [56], underlining the regulatory role that could be taken up by an epiphyte with respect to this.

Our findings have implications for strategies that seek to apply PGPB and, specifically, ACC deaminase expressing microorganisms within agriculturally relevant biologicals [9, 10, 22, 57]. Laboratory tested PGPR often fail to deliver the expected results in field trials. To date, the field suffers from a lack of applicability. The reasons are diverse, but they likely include using non-native host-microbe networks and artificial environments for testing [58–60]. Another important factor limiting applicability of “lab-strains” may be the high microbial diversity in soils and especially in rhizosphere communities, which negatively impacts the potential for survival and establishment of non-selected or not optimally adapted players and latecomers into these environments [61, 62]. While, with a few exceptions, the phyllosphere microbiota is similarly difficult to perturb once established [62] there are some key differences: the phyllosphere is highly accessible to manipulation [63] both at an early stage and during plant development. Practical manipulations of the microbiota could be more successful if applied early in a host’s life cycle or to newly emerging tissues when the microbial community is still developing [62]. The accessibility of the phyllosphere throughout a plant’s life cycle is key to successfully introducing beneficial bioagents. It allows for interventions to reconstitute and maintain host-microbiota homeostasis at any point in time during cultivation. Therefore, it seems more achievable to exploit the growth promoting and stress alleviating effects of ACC hydrolyzing bacteria in the field through inoculation via the leaves. Of course, the induction of shifts in the leaf microbial community and the resulting change in overall microbe-microbe interactions and their consequences are going to have to be considered. While generally reconstituting natural and healthy microbiomes is certainly a very promising strategy to ensure high productivity in agriculture, we speculate that the impact of treatment with a single PGPB may be strongest if applied through the phyllosphere early in the plants’ life cycle.

## Conclusion

ACC hydrolysis by PGPB has proven to be a powerful tool for alleviating stress and for the promotion of plant growth under laboratory conditions. To transfer these tools into application we need to think about strategies for their successful implementation. Manipulation of plant ethylene pools seems to work through root and, importantly, through shoot application, for which we provided ample evidence. Making use of the accessible nature of above ground plant tissues and exploiting pioneer effects on developing plantlets may help to realize the potential of microbe-assisted crop production.

## Methods

No statistical methods were used to predetermine sample size. The experiments were randomized and investigators were not blinded to allocation during experiments and outcome assessment.

### Long term tomato growth assays in soil

#### Bacterial culture and plant inoculation

Strain Msb3 was grown from cryo-stock in liquid culture overnight at 28 °C in tryptic soy broth (TSB) (*Carl Roth GmbH & Co., KG*). It was harvested at an OD_600_ of 0.6 and washed three times with 1 × PBS. Bacterial suspensions of an OD_600_ of 0.1 in PBS were used for plant inoculations, which corresponds to approximately 3 × 10^6^ colony forming units (CFUs) ml^–1^. Seedlings were dipped into the suspension for 10 seconds. They did not retain significant amounts of liquid (>100 µl) when they were removed.

#### Plant growth conditions

Seeds of tomato (*Lycopersicum esculentum* cv. Moneymaker) were surface sterilized by immersion in 70% (v/v) ethanol for 5 min followed by incubation in 2.8% NaClO for 10 min and subsequently washed with sterile distilled water for 30 min. The treated seeds were placed onto Murashige & Skoog (MS) agar medium including B5 vitamins (*Duchefa Biochemie B.V, Haarlem, Netherlands*) and germinated in the dark at 22 °C. After seven days seedlings were exposed to light and after 10 days uniform seedlings were selected and inoculated with bacteria. The seedlings were dipped into bacterial suspensions of either live or heat killed bacteria and were subsequently transferred to a 300 ml pot containing 60 g of hot vapor treated garden soil (150–600 mg l^–1^ N, 150–600 mg l^–1^ P_2_O_5_, 200–900 mg l^–1^ K_2_O, 150 mg l^–1^ Mg, pH = 5–6.5) (*Franz Kranzinger GmbH, Straßwalchen, Austria*). All pots were randomly placed in a growth chamber for 60 days at 22 °C, a photoperiod of 16 h (100–130 PPFD, μmol m^–2^ s^–1^ at the ground level), and relative humidity of 65%. After 25 and 45 days one third of all pots was fertilized with 20 mL 0.1% WUXAL Super (*Hauert Manna Düngerwerke GmbH*, *Austria*) consisting of 8% N (2.3% NO_3_^–^, 3.7% NH_4_^+^, 2% Urea), 8% P_2_O_5_, 6% K_2_O and trace elements in distilled water (conductivity 0.51 mS).

#### DNA extraction

DNA was extracted from approximately 50 mg of frozen leaf tissue. The tissue was sampled from emerging leaves next to the apical meristem. Homogenized tissue was mixed and washed with alkaline PVPP buffer (Tris–HCl, pH=9.5, 50 mM; EDTA, 10 mM; NaCl, 4 M; CTAB, 1%; PVPP, 0.5%; 2-mercaptoethanol, 1%) and the supernatant was used for further DNA extraction following a standard P/C/I protocol. Chloroform phase separation was repeated three times to enhance phenol residue removal. DNA was precipitated in 0.3 M NaOAc and 70% EtOH at −20 °C and stored in ddH_2_O at −20 °C.

#### Quantitative PCR

Quantitative PCRs (qPCRs) were performed using the iTaq Universal SYBR Green Supermix (*Bio-Rad Laboratories, Inc., CA, USA*) on a CFX Connect Real-Time PCR Detection System (*Bio-Rad*). Three technical replicates of each biological replicate of every experiment were measured. Gene copies of the *gyrB* gene were quantified using the species-specific primer pair gyrBqMsb3_F/R targeting a 131 bp region within the *gyrB* gene of Msb3. Absolute copy numbers in the samples were determined using a standard (10 to 10^8^ copies/µl) produced via a full length *gyrB* amplicon generated through an end-point PCR on DNA extracted from an Msb3 pure culture using the primer pair gyrB_F/R. Information concerning end-point PCR settings can be found the Supplementary Material and a list of primers used in the present study in Supplementary Data 1.

### In vitro culture assays in tomato

#### In vitro culture setup

We set up in vitro cultures to sustain tomato growth for several weeks within sterile glass jars in growth medium. As a growth medium we used 15–50 ml 1 × MS agar medium within 200 ml jars, which were sealed with Magenta B-Cap Jar Lids (*bioWORLD, OH, USA*) and parafilm. Tomato seeds were sterilized and germinated on 12 × 12 cm MS agar plates as described before. Uniform seedlings were selected and, after application of treatments, individually transferred to glass jars, which were randomly placed in growth chambers with a photoperiod of 16 h at 25 °C during the day and 18 °C during the night and a relative humidity of 60%.

#### Bacterial culture and plant inoculation

Strains Msb3 and PsJN were grown from cryo-stock in liquid culture overnight at 28 °C in tryptic soy broth (TSB) (*Carl Roth*), shaking at 250 rpm. Bacteria were harvested at an OD_600_ of 0.6 and washed three times with 10 mM MgCl_2_. Bacterial suspensions of an OD_600_ of 0.1, 0.01 or 0.001 in MgCl_2_ were used for plant inoculations, which corresponds to approximately 3 × 10^6^, 3 × 10^5^ and 3 × 10^4^ CFUs ml^–1^, respectively. Seedlings were dipped into the suspension for 10 seconds. They did not retain significant amounts of liquid (>100 µl) when they were removed. Dilutions with OD_600_ of 0.1 were used for the timeseries experiment, those with an OD_600_ of 0.01 were used for growth promotion assays and those with an OD_600_ of 0.001 for monitoring early infection dynamics.

#### Monitoring early infection dynamics via CLSM

Seedlings were inoculated with a highly diluted bacterial suspension (OD_600_ of 0.001) or not at all and grown in triplicates for 24 h, 48 h, and 72 h. Seedlings were washed in 1 × PBS (pH = 7.2) for 30 s after harvest. Small sections of the topmost leaflets or the primary root were used for preparation of microscopy samples.

Fluorescent in situ hybridization was conducted as described previously [31]. Images were taken on a confocal laser scanning microscope (Leica DMRE SP5) (*Leica Microsystems GmbH, Wetzlar, Germany*). Detailed information can be found in the Supplementary Material.

#### Weekly monitoring experiment

Seedlings were inoculated with suspensions of Msb3, PsJN or not at all. 60 uniform seedlings were selected and 20 were used for each treatment group. All jars were placed randomly into the growth chamber. At each timepoint five random jars within each treatment group were selected. When plants did not survive being transferred or adhered to the glass and succumbed within the first few days they were removed. At least three replicates could be recovered for each timepoint. Samples were taken once a week: 7 DPI, 14 DPI, 21 DPI and 28 DPI. At the first timepoint we only took samples of the shoots but proceeded to taking both root and shoot samples for all other timepoints. The entire shoot or root system was used for DNA extractions. Further information with detailed instructions for DNA extractions can be found in the Supplementary Material.

#### Quantitative PCR

Quantitative PCRs (qPCRs) were performed using the Luna Universal qPCR Master Mix (*New England Biolabs, MA, USA*) on a CFX Connect Real-Time PCR Detection System (*Bio-Rad*). Three technical replicates of each biological replicate of every experiment were measured. Gene copies of the *gyrB* gene were quantified as described in the previous section.

#### *Paraburkholderia dioscoreae* Msb3 *acdS* knockout construction

The unmarked deletion mutant *Paraburkholderia dioscoreae* Msb3Δ1965167-1966395 on chromosome II (Msb3ΔacdS) was constructed based on a genetic system developed for *Burkholderia* spp. and the suicide vector pMo130 [38].

#### Knockout suicide vector pAJ101 construction

The empty vector backbone pMo130 was obtained from Addgene (*Addgene, MA, USA*) with *E. coli* JM109, which was grown at 30 °C in 50 µg/ml kanamycin. Plasmid DNA was isolated using the PureYield Plasmid Miniprep System (*Promega, WI, USA*). pMo130 was linearized using the restriction enzymes NheI-HF and HindIII-HF (*New England Biolabs*). One-kb regions for homologous recombination flanking the Msb3 *acdS* gene were amplified using primers acdS_US_F1-R1 and acdS_DS_F1-R1 (Supplementary Data 1). The resulting PCR products were used as an input for another round of amplification with primers containing restriction motives. This amplification was done with primers that amplify the upstream (US) fragment flanked by NheI and BglII restriction sites, as well as the downstream (DS) fragment flanked by BglII and HindIII restriction sites. Resulting PCR products were purified and digested with the respective enzymes and subsequently ligated with Quick Ligase (*New England Biolabs*). The resulting 2-kb fragment was ligated into the linear vector backbone. The vector pAJ101 containing the recombination flanks was heat shocked into *E. coli* WM3064. *E. coli* WM3064 containing pAJ101 was maintained at 31 °C on LB containing 50 μg/ml kanamycin and 0.3 mM diaminopimelic acid (DAP).

#### Conjugative transfer of pAJ101 into Msb3

For biparental mating, *E. coli* WM3064 containing pAJ101 was grown as above and Msb3 was grown in 1 × LB without antibiotics. Msb3 was harvested at an OD_600_ > 0.7 and *E. coli* WM3064 at an OD_600_ of 0.5. Each strain was washed separately two times with 1 × LB medium, then they were mixed at ratios 1:1, centrifuged and resuspended in approximately 1/10 the volume and plated in a single pool on LB agar containing 0.3 mM DAP and were grown at 28 °C overnight. Exconjugants were streaked onto 1 × LB or M9 minimal medium with glucose plates lacking DAP and 100 μg/ml kanamycin to select for bacteria that had completed the first crossover. First crossover strains were purified three times by re-streaking.

#### Resolution of pAJ101 integration and knockout strain purification

To resolve the integration of pAJ101, first crossover strains were grown in LB medium containing 100 μg/ml kanamycin. Subsequently they were plated on YT medium containing 10% sucrose (yeast tryptone medium: 10 g/l tryptone, 5 g/l yeast extract, 100 g/l sucrose, 1% agar). Colonies were picked into the same liquid medium and grown once. The resulting strains were screened by PCR for deletion of *acdS* using primers acds_KO_control_F/R and acdSF1/R1 (Supplementary Data 1). To ensure strain purity, PCR primers were designed to amplify 200 bp of the gene itself and another set to sequence across the deleted region. Msb3 wt DNA was used as control. These strains were subsequently plate-purified at least three times on M9 minimal medium with glucose without antibiotics.

### Msb3ΔacdS growth promotion assays in tomato

#### Setup

We inoculated 7-day old tomato seedlings with suspensions of Msb3 wildtype, the *acdS* deficient strain Msb3ΔacdS and a non-inoculated control. After another week of growth, we determined phenotypic parameters.

#### Bacterial and plant cultures and inoculation

To measure the effect of different Msb3 mutants on tomato development and phenotype, growth promotion assays were conducted using the in vitro growth system for tomato as well as a non-gnotobiotic system in soil. For some in vitro experiments, the plant culture medium was supplemented with 400 nM ACC. A bacterial suspension of an OD_600_ of 0.01 in 10 mM MgCl_2_ was used for plant inoculation. Msb3ΔacdS was grown exactly like Msb3. For in vitro experiments, the seedlings were dipped into the suspensions as described before.

For non-gnotobiotic experiments, untreated tomato seeds were germinated on paper soaked in tap water in the dark. Uniform seedlings were either dipped into the bacterial suspension prior to transplanting or sprayed with the bacterial suspension afterwards depending on the mode of inoculation. Seedlings were transplanted into non-treated garden soil in 5 × 5 cm pots. The soil was covered with two layers of 0.22 µm pore size Whatman filter paper while spraying. Every seedling received exactly one spray pump, consisting of approximately 1 ml. The plants were placed randomly inside a growth chamber as described before and were watered daily using tap water.

#### Growth promotion assay

Inoculated tomato seedlings were grown for seven days in a growth chamber under conditions previously outlined. After seven days, all plantlets were removed from their containers, and for in vitro assays, the number of side roots, their cumulative length, the length of the primary root, and that of the hypocotyl were determined. Side root number and length were determined manually. Primary root and hypocotyl lengths were determined via image analysis.

For non-gnotobiotic assays, we could only unambiguously determine hypocotyl length via image analysis, as we could not exclude damaging the root while pulling it out of the soil. Instead, we measured the weight of the seedlings.

#### Root and shoot image analysis

Plants were imaged using a fixed Nikon camera and a scale was included in each image. Primary root length elongation and hypocotyl length was measured using ImageJ.

#### Phenotypic analyses

Phenotypic parameters were compared across the no bacteria, Msb3 and Msb3ΔacdS treatments using a two-sided ANOVA model. Normal distribution was assumed. Differences between treatments were indicated using the confidence letter display derived from the Tukey’s *post hoc* test implemented in the package *agricolae*.

### Measuring RGI in *Arabidopsis*

#### Experimental design

This experiment included the following treatments: (i) no bacteria, (ii) *P. dioscoreae* Msb3 and (iii) Msb3ΔacdS. Each treatment was repeated in two separate agar plates with four *Arabidopsis* seedlings per plate (*n* = 8).

#### Bacterial culture and plant inoculation

All strains were grown in separate tubes to an OD_600_ of 0.6, washed with MgCl_2_ and OD_600_ was adjusted to 0.01 before spreading 100 μl of each bacterial treatment onto 12 × 12 cm Johnson Medium (JM) agar plates containing 100 nM ACC. Plants were placed onto the vertically incubated plates.

#### In vitro plant growth conditions

All seeds were surface-sterilized with 70% bleach, 0.2% Tween-20 for 8 min, and rinsed three times with sterile distilled water. Seeds were stratified at 4 °C in the dark for three days. Plants were germinated on vertical square 12 × 12-cm agar plates with JM containing 0.5% sucrose, for seven days. Then, four plants were transferred to each of the agar plates inoculated with bacteria. Plates were placed in randomized order in growth chambers and grown under a 16 h dark/8 h light regime at 21 °C day/18 °C night for seven days.

#### Primary root elongation analysis

Differences between treatments were indicated using the confidence letter display derived from the Tukey’s *post hoc* test from an ANOVA model.

#### Preparation of binarized plant images

To present representative plant images, we increased contrast and subtracted background in ImageJ, then cropped the image to select representative roots. Neighboring roots were manually erased from the cropped images.

### Re-cultivation experiments

#### Construction of fluorescently tagged *Paraburkholderia* strains

We created fluorescently tagged bacterial strains that were resistant to kanamycin via stable chromosomal insertions via a Tn5 transposon-based delivery system [64]. Both plasmids were delivered in *E. coli* strain S17-1 that allows conjugation of the mobilizable plasmids into other bacteria. *E. coli* strain S17-1 was grown in 1 × LB containing 50 µg/ml ampicillin at 30 °C to an OD_600_ of 0.3. Recipients were grown in 1 × LB without antibiotics to an OD_600_ of around 0.7. Each strain was washed separately two times with 1 × LB medium, then they were mixed at ratios 1:1, centrifuged and resuspended in approximately 1/10 the volume and plated in a single pool on 1 × LB agar. The mixture was incubated at 28 °C overnight. Exconjugants were streaked onto M9 minimal medium agar plates containing glucose or malic acid as sole carbon source and 100 µg/ml kanamycin. After three to five days the colonies that appeared were re-streaked on the same media. Three rounds of re-streaks were performed to make sure the strains were pure.

#### Screening of fluorescently tagged strains

Fluorescently tagged strains were screened for their ability to grow normally in rich medium. If a strain displayed impaired growth it was not used in subsequent re-cultivation experiments. Fluorophore expression was checked on a GelDoc Go Imaging System on a UV/Stain-Free Tray (Bio-Rad). If there was no growth deficiency and proper fluorophore expression one eGFP2 and one mScarlet expressing clone of each strain was chosen for downstream experiments. The GelDoc Go Imaging System was equipped with filters for fluorescence of various commercial dyes. A culture of Msb3 wt was used as negative control and the *E. coli* donor strains were used as positive controls to create reproducible settings. Of each plate one brightfield image was obtained (exposure for 0.5 s) as well as one image through a filter for Atto488 (0.3 s) and one through a filter for Cy3 (4 s).

#### Re-cultivation assay setup

Re-cultivation experiments were conducted using the in vitro growth system for tomato. Fluorescently tagged strains were grown to an OD_600_ of 0.6 in TSB containing 100 µg/ml kanamycin. Cells were washed twice in 10 mM MgCl_2_. Plants were inoculated as described before. Plants were grown for seven days after inoculation. Upon harvest, the plant was removed from the container, shoot and root systems were separated and residual agar was removed from the roots by washing with glass beads in 0.4% NaCl. Using sterile mortar and pestle the tissue was carefully ground in 1 ml 0.4% NaCl. The liquid was recovered and used to create serial dilutions. 5 µl were plated onto M9 minimal medium agar containing 20 g/l glucose and 100 µg/ml kanamycin in a dropwise fashion. After 3 days the plates were imaged as outlined above and the resulting images were used to quantify CFUs.

## Supplementary information

Supplemental Material

Supplemental Table 1

Supplemental Table 2

## Data Availability

All data needed to evaluate the conclusions presented here have been included in the paper and/or the Supplementary Materials. Additional information or materials related to this article can be supplied upon correspondence.
