# Supplementary material for: Phyllosphere symbiont promotes plant growth through ACC deaminase production
Source: ISME J. 2023 Jun 1;17(8):1267–77. doi: 10.1038/s41396-023-01428-7 (PMC10356760; doi:10.1038/s41396-023-01428-7)
Supplement: Supplementary file 1 — Supplemental Material [file 41396_2023_1428_MOESM1_ESM.pdf]

Supplementary Material

to

Phyllosphere symbiont promotes plant growth through ACC deaminase production

Johannes Herpell[1], Ajtena Alickovic[1], Bocar Diallo[1], Florian Schindler[1] and Wolfram Weckwerth[1,2]*

1. Molecular Systems Biology Division, Department of Functional and Evolutionary Ecology, University of Vienna, Djerassiplatz 1, 1030 Vienna, Austria.

2. Vienna Metabolomics Center, University of Vienna, Djerassiplatz 1, 1030 Vienna, Austria.

* Correspondence to: wolfram.weckwerth@univie.ac.at

Supplementary Figures

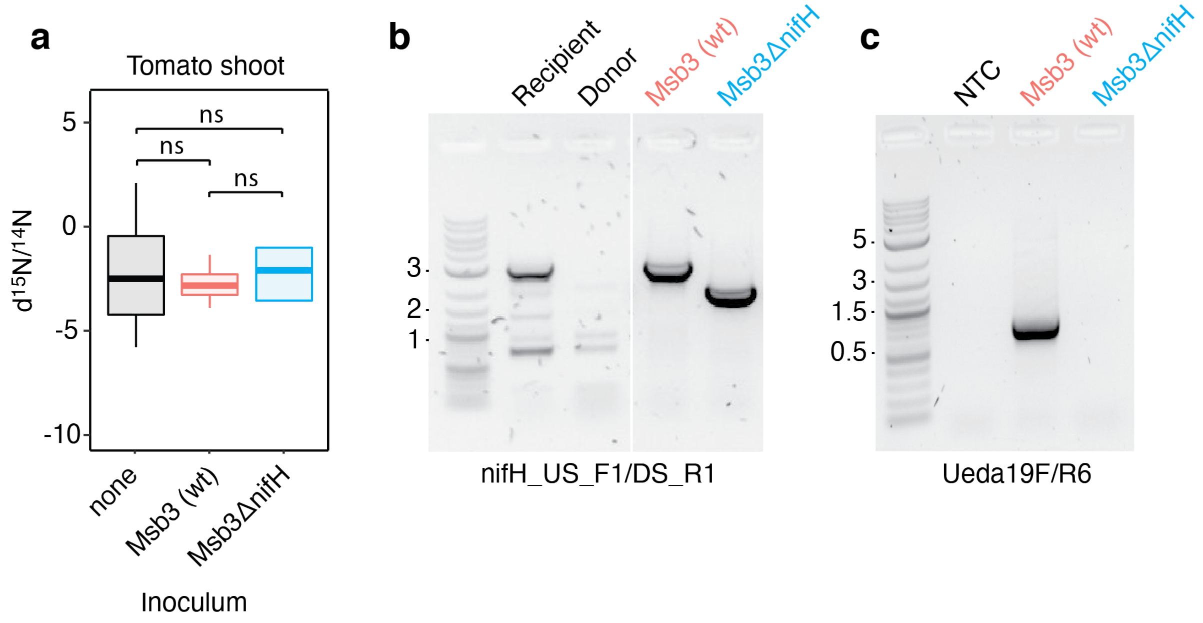

Supplementary Figure 1 | $^{15}N_2$ labelling experiments in the Tomato phyllosphere with Msb3 wt and Msb3ΔnifH. a, Tomato seedlings were grown *in vitro* in Schott-bottles sealed with rubber lids. Before labelling, the plants were inoculated with either no bacteria (control), Msb3 wildtype or the *nifH* deficient mutant Msb3ΔnifH. The atmosphere inside the bottles was gradually replaced with an artificial atmosphere containing at least 20% $^{15}N_2$ labelled gas. The pressure inside the bottles was kept at atmospheric pressure. After two days the atmosphere was exchanged again. After four days the plants were harvested, dried and the d^{15}N/^{14}N ratio was determined via isotope ratio mass spectrometry (IR-MS). No significant differences between the treatment groups could be detected (n=4;4;3). Significance was determined between treatments via an ANOVA and Tukey post hoc test. **b**, PCR results after amplification of the 3 kb fragment between the primers nifH_US_F1 and nifH_DS_R1, flanking the Msb3 *nifH* gene. The conjugational donor (*E. coli* WM3064) of the knockout vector was used as a negative control. Msb3 (wt) was used as a recipient for the vector and as positive control. After allelic exchange the *nifH* knockout produces a band 1012 bp smaller than the wildtype. **c**, Amplification of the *nifH* gene using primers Ueda19F [1] and R6 [2]. A no template control (NTC) functioned as negative control, Msb3 wt DNA as a positive control. Using DNA of the *nifH* knockout as input does not result in amplification.

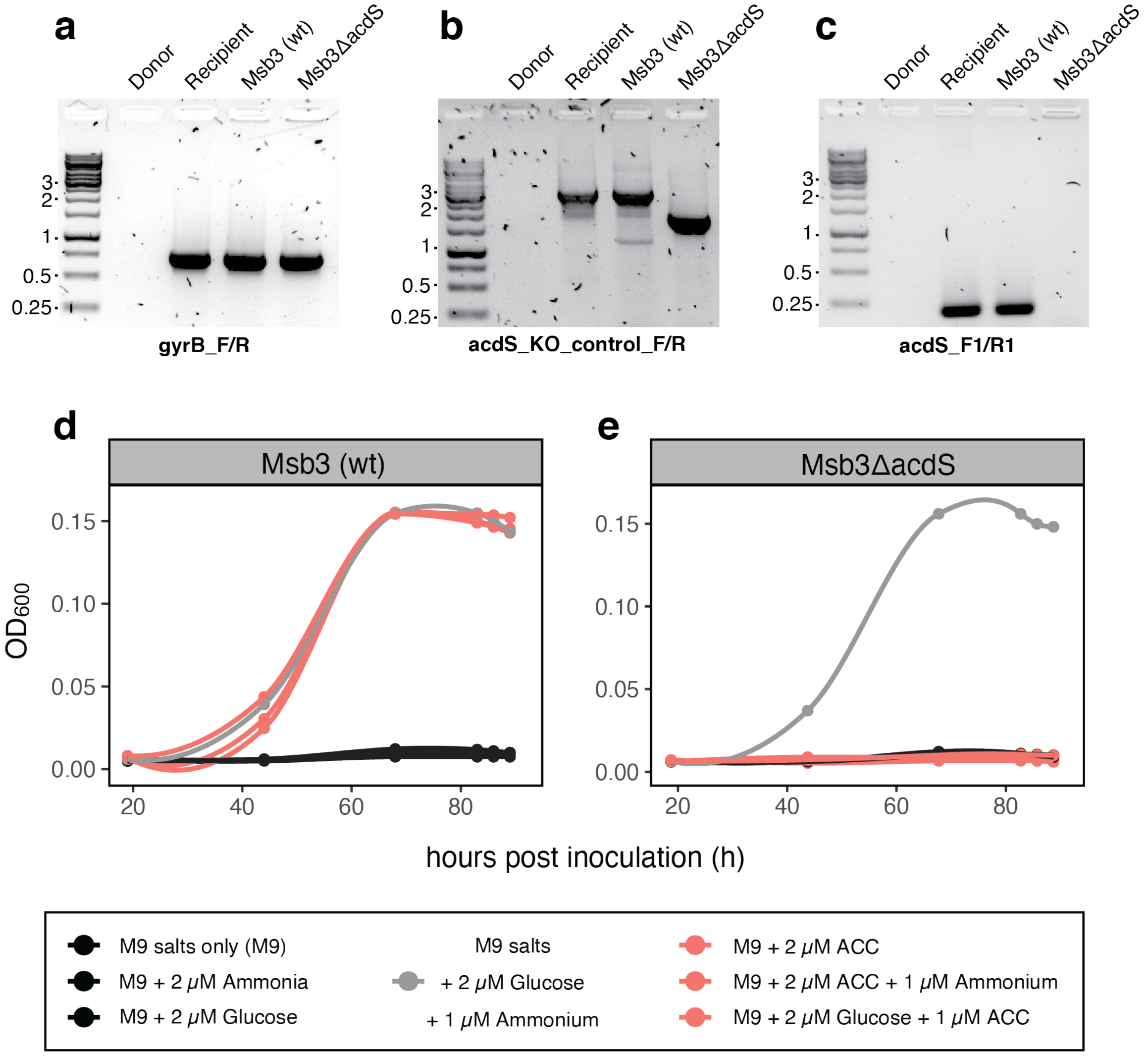

Supplementary Figure 2 | ACC deaminase deficiency of Msb3ΔacdS. a, PCR results after amplification with the primer pair gyrB_F/R, which is specific to *Burkholderia* spp. *sensu lato* species. The *E. coli* donor, strain WM3064, without pAJ101 does not get picked up by the primer. The recipient, strain Msb3 before conjugation, gives a clear band. Msb3 (wt) DNA was used as a positive control. After conjugation and allelic exchange, the exconjugant Msb3ΔacdS was still detected. Sequencing indicated that the gyrase B fragment amplified was 100% identical to that of the Msb3 wt. **b**, PCR on the same set of organisms using a forward primer binding >1kb upstream of *acdS* and a reverse primer binding >1kb downstream of *acdS*. In Msb3 wildtypes the resulting amplicon is approximately 3 kb, after successfully knockout of *acdS* the fragment is 1228 bp smaller. **c**, as a final control we designed primers amplifying 193 bp of the Msb3 *acdS* gene itself. In Msb3ΔacdS it can no longer be amplified. **d** and **e**, growth of strains Msb3 (**d**) and the *acdS* deficient mutant (**e**) in M9 minimal medium containing different nutrient sources. Black lines represent negative controls in which an essential nutrient source is missing (n=1). The grey line represents a positive control that contains both a source of carbon and nitrogen (n=1). Red lines represent conditions in which the media were supplemented with ACC instead of either carbon (n=3), nitrogen (n=3) or both (n=3).

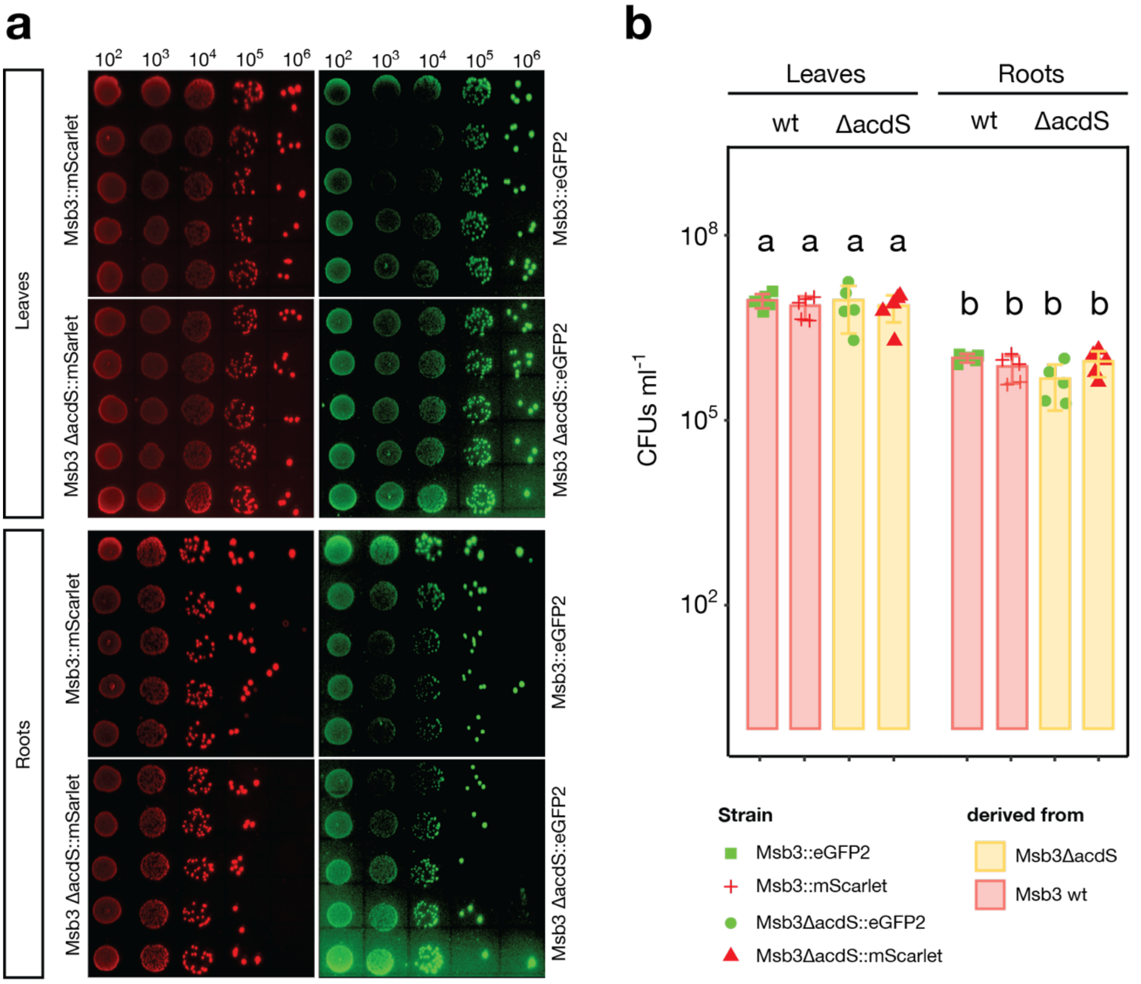

Supplementary Figure 3 | Re-cultivation of Msb3 wt and *acdS* deficient mutant from Tomato leaves and roots 7 DPI. a, Re-cultivation assays showing serially diluted plant extracts plated onto M9 minimal medium agar containing glucose as carbon source and kanamycin. Each column represents a tenfold serial dilution as indicated on top of each column. Each row represents a biological replicate inoculated with one of four mutants: Msb3 wildtype derivative strains Msb3::eGFP2 or Msb3::mScarlet or Msb3ΔacdS derivative strains Msb3ΔacdS::eGFP2 or Msb3ΔacdS::mScarlet. The upper panels show CFUs re-cultivated from leaves, the lower ones from roots. Plant extracts were prepared using 1 ml of 10 mM $MgCl_2$. 5 µl of each serial dilution were used for plating. **b**, results of **a** after quantification of CFUs ml^{-1} in the original extract. Serial dilutions of 10^5 were used to calculate the final values. In our assay the *acdS* KO does not affect the capacity of strain Msb3 to colonize leaf or root tissue. Like in qPCRs, Msb3 is by an order of magnitude more abundant in leaves compared to roots.

Supplementary Methods

DNA extractions

Nucleic acid extractions were carried out as outlined in Oliveira et al. [3]. This method relies on the removal of RNA from the nucleic acid pool and resulted in much better downstream application performance regarding DNA. Samples were kept on ice at all times to prevent degradation and all centrifugation steps were carried out at maximum speed at 4°C. Plant tissue was placed in 2 mL tubes and 400 µL of extraction buffer (0.4 M LiCl; 0.2 M Tris-HCL, pH 8.0; 25 mM EDTA, pH 8.0; 1% SDS; 4.7% β-mercaptoethanol) was added. The samples were macerated with small sterile pestles directly inside of the tubes. Pre-cooled chloroform:isoamylalcohol (24:1) was added to the samples and the mixture was vortexed and centrifuged and the upper phase was transferred to a new 1.5 mL tube. 500 µL of basic phenol (pH 8.0) was added and the tubes were mixed thoroughly. 200 µL pre-cooled chloroform:isoamyl alcohol was added and tubes were vortexed and then centrifuged. The supernatant was transferred to a fresh 1.5 mL tube and 1/3 of the sample volume of an 8 M LiCl solution was added. The tubes were mixed by repeated inversion. RNA was precipitated at -20°C for at least one hour or up to 16 h, overnight. After precipitation the samples were centrifuged for 30 min. The tube containing the pellet can be used for isolation of total RNA. The supernatant was used for isolation of DNA.
DNA was precipitated with isopropanol at -20°C. The pellet was washed twice with ethanol, first with 70% followed by 95% EtOH. After washing, the samples were dried for 30 min at room temperature and solubilized in 0.1 × TE (pH 8.0). DNA was stored at -20°C.

End-point PCR

DNA was amplified using Phire Hot Start II Polymerase (*Thermo Fisher Scientific*) in 20 µl reaction mixtures consisting of 11.7 µl sterile ddH_2O, 4 µl 5 × Phire Green Reaction Buffer (1.5 mM $MgCl_2$ final concentration), 0.4 µl dNTP Solution Mix (10 mM of each dNTP), 0.3 µl Phire Hot Start II Polymerase and 1 ml 10 mM forward and 1 ml 10 mM reverse primer as well as 1 µl template DNA (1 ng/µl) or a colony lysed in 10 µl sterile ddH_2O. PCR products were purified using a Monarch® PCR & DNA Cleanup Kit or Monarch® DNA Gel Extraction Kit (*New England Biolabs*). Primers were purchased from Microsynth (*Microsynth AG, Balgach, Switzerland*). A list of all the primer sequences used in this study and their properties is presented in **Supplementary Data 1**.

CLSM/FISH

Plant samples were fixed in 2% paraformaldehyde in 1 × PBS (pH = 7.2) for 1 h and subsequently washed in PBS, twice. FISH analysis was conducted as follows:

Treatment with lysozyme solution (1 mg ml^{-1} in PBS) was applied for 10 min at 37°C and subsequently the samples were dehydrated in a stepwise ethanol series (25, 50, 75, and 99.9%; 15 min each step). Double labeling of oligonucleotide probes for FISH (DOPE-FISH) was performed with probes labeled at both the 5′ and 3′ end positions with the respective fluorophore (*Microsynth*). A *Burkholderia* sensu lato specific probe Burkho [4] coupled with Cy3 was used for detection of the bacteria. Hybridization was carried out at 46°C for 2 h or overnight within Eppendorf tubes containing the hybridization solution (20 mM Tris–HCl pH 8.0, 0.01% w/v SDS, 0.9 M NaCl, 10 ng μl^{-1} of the probe 30% formamide) and the respective plant sample. Post-hybridization was conducted at 48°C for 30 min with a pre-warmed post-FISH solution containing 20 mM Tris–HCl pH 8.0, 0.01% SDS, 5 mM EDTA pH 8.0 and 0.102 M NaCl. Samples were rinsed with ice cold distilled water before air drying in the dark. Nucleic acids were stained by immersion of the specimen in 10 × SYBR® Safe (*Thermo Fisher Scientific*) in 1 × PBS for 10 min. Excess dye was removed through two additional washing steps. The samples were mounted in VECTASHIELD® Antifade Mounting Medium (*Vector Laboratories, Inc., Newark, CA United States*) and then observed under a confocal microscope.
Images were taken on a confocal laser scanning microscope (CLSM) (Leica DMRE SP5) (*Leica Microsystems*). A white light laser was used for excitation of fluorescence signals (SYBR® Safe: ex/em 410/520-540 nm; “chlorophyll autofluorescence”: ex/em 550 or 555/674-716 nm; Cy3: ex/em 550/560-590 nm). The scanning speed was 100 Hz and a 20× or 63× glycerol immersion objective was used for all images. Images were taken sequentially to prevent leaking of fluorescent signals into other channels. Stacks of 15 to 25 optical sections per series were recorded to cover a large range and the fluorescence data was combined in maximum projections using the Leica Application Suite (LAS) X (*Leica Microsystems*) software platform. Adaptation of brightness and contrast of all images to better suit print and screen representation was conducted through ImageJ version 1.52q [5].

Paraburkholderia dioscoreae Msb3 *nifH* knockout construction and ^{15}N labelling of Tomato

This section relates to **Supplementary Figure 1**. The *nifH* knockout was constructed as previously described for *acdS* using primers listed in **Supplementary Data 1** and conjugational donor strains and plasmids listed in **Supplementary Data 2**.
Tomato seedlings were grown and inoculated as described in *in vitro* growth assays in Tomato. We used 100 ml Schott-bottles instead of glass jars, which were sealed with rubber lids. Plants were inoculated with either no bacteria (control), Msb3 wildtype or the *nifH* deficient mutant Msb3ΔnifH. The atmosphere inside the bottles was replaced with an artificial atmosphere containing at least 20% $^{15}N_2$ labelled gas via removal of air through a syringe on one side and resupply of labelled air through another syringe simultaneously. Like this, the pressure inside the bottles was kept constant at all times. After two days the atmosphere was exchanged again. After four

days the plants were harvested, dried at 50°C in a drying oven and the $d^{15}N/^{14}N$ ratio was determined via isotope ratio mass spectrometry (IR-MS) on an isotope ratio mass spectrometer (IRMS; Delta-PLUS, Thermo Finnigan) coupled with an interface (ConFloIII, Thermo Finnigan) to an elemental analyzer (EA 1110, CE Instruments).

In vitro growth of Msb3ΔacdS and Msb3 on ACC
This section relates to **Supplementary Figure 2**.

Both strains were grown from cryostock in TSB. An overnight culture of each was washed thoroughly with 10 mM $MgCl_2$, at least three times. Washed cells functioned as an inoculum of M9 minimal media with various sources of carbon or nitrogen. As negative controls we included following conditions: M9 salts only; M9 salts with 2 µM glucose; M9 salts with 2 µM ammonia. As positive control we used M9 minimal medium supplemented with both 2 µM of glucose as carbon source as well as 1µM of ammonia as nitrogen source. To test growth on ACC we tested three different conditions: ACC as carbon source (M9 salts, 2 µM ACC, 1 µM ammonia); ACC as nitrogen source (M9 salts, 1 µM ACC, 2 µM glucose); ACC as both carbon and nitrogen source (M9 salts, 2 µM ACC). We inoculated these media with a very small number of cells to prevent cell debris from functioning as nutrient source (OD_{600} < 0.001). Growth was monitored over a period of 4 days via OD_{600} measurements.

References

1. Ueda T, Suga Y, Yahiro N, Matsuguchi T. Remarkable N2-fixing bacterial diversity detected in rice roots by molecular evolutionary analysis of nifH gene sequences. *Journal of Bacteriology* 1995; **177:** 1414-1417.

2. Marusina AI, Boulygina ES, Kuznetsov BB, Tourova TP, Kravchenko IK, Gal'chenko VF. A System of Oligonucleotide Primers for the Amplification of nifH Genes of Different Taxonomic Groups of Prokaryotes. *Microbiology* 2001; **70:** 73-78.

3. Oliveira R, Viana A, Reátegui A, Vincentz M. An efficient method for simultaneous extraction of high-quality RNA and DNA from various plant tissues. *Genetics and Molecular Research* 2015; **14:** 18828-18838.

4. Hogardt M, Trebesius K, Geiger AM, Hornef M, Rosenecker J, Heesemann J. Specific and rapid detection by fluorescent in situ hybridization of bacteria in clinical samples obtained from cystic fibrosis patients. *Journal of clinical microbiology* 2000; **38:** 818-825.

5. Schneider CA, Rasband WS, Eliceiri KW. NIH Image to ImageJ: 25 years of image analysis. *Nat Methods* 2012; **9:** 671-675.
